# A Numerical Method of Aligning the Optical Stacks for All Pixels

**DOI:** 10.3390/s23020702

**Published:** 2023-01-08

**Authors:** Jae-Hyeok Hwang, Yunkyung Kim

**Affiliations:** 1 Department of ICT Integrated Safe Ocean Smart Cities Engineering, Dong-A University, Busan 49315, Republic of Korea; 2 Department of Electronic Engineering, Dong-A University, Busan 49315, Republic of Korea

**Keywords:** CMOS image sensor, optical stacks, chief ray angle, numerical method, FDTD simulation

## Abstract

Reducing performance verification time is significant in product launch and production costs. This is especially true because aligning the optical stacks of off-axis pixels is a time-consuming task, but it is important to maintain sensitivity. In this paper, a numerical method to align the optical stacks of off-axis pixels is suggested in order to reduce performance test time. The components of the numerical method are the optical stack height, refractive index, and chief ray angle in order to calculate the optical stacks’ optimal shift distance. The proposed method was investigated to confirm effectiveness by using optical simulation. The sub-micron backside illumination (BSI) pixels were simulated, having 2 × 2 microlens, quad-color filter array, and in-pixel deep trench isolation (DTI). Moreover, the proposed method was evaluated for various pixel pitches, microlens shapes, and CRAs. As a result, the optical stacks were optimized by using the numerical method and validated via optical simulation. Therefore, the proposed numerical method is expected to help reduce the time and cost.

## 1. Introduction

The camera module vertically consists of image sensors and module lenses for image formation. To eliminate aberrations and provide various fields of view, the module lenses are designed in multiple layers. For a clear and vivid image, light must pass through the module lenses to each pixel in all positions of the image sensor without loss. The incident light entering each pixel has different chief ray angles (CRAs) according to the pixel position in the image sensor [1]. The CRA indicates the chief angle of light from the module lens to the middle of the pixel. The on-axis pixels at the center of the image sensor are defined as CRA 0°. Thus, the CRAs of off-axis pixels are more than 0°. In other words, the incident light obliquely enters the off-axis pixel, excepting CRA 0°. Moreover, the CRA increases as pixels are located at the image sensor’s edges. Especially, the CRA is increased in imaging devices such as mobile cameras and medical imaging applications that have a tendency of shortening focal lengths and large sizes of image sensors. In the case of the trend for mobile cameras, the size of the image sensor has been getting larger for high resolution images [2]. As large image sensors have been widely used, the number of module lenses have been continuously increasing to eliminate aberration [3]. On the other hand, the camera module thickness is limited and causes a camera bump due to the size of the smartphone [4]. Therefore, the CRA at the edge of the image sensor is almost 35° due to the limited focal length between the camera module and the image sensor [3,5]. As the CRA increases, the oblique incident light affects the pixel’s sensitivity [6]. Therefore, CRA is one of the significant optical characteristics to consider in image sensors.

The optical pixel stacks are vertically composed of a microlens, color filter (CF), a passivation layer (PL), and so on. While the oblique incident light is entering the photodiode, the optical stacks of the adjacent pixel are able to absorb and reflect the unwanted light [7,8]. Optical loss by oblique incident light is inevitable at the off-axis pixels. Therefore, light transmitted to each photodiode is reduced due to increased light loss as the CRA increases. Several pixel structures have been suggested in order to resolve optical loss at the off-axis in the image sensor. Microlenses having different shapes were suggested by changing the curvature of microlens according to CRA [9]. Shapes of the microlens were individually fabricated by using a mask with a gradation pattern. When implementing an individual microlens, the focal point’s position was able to change toward the photodiode. However, changing the curvature of every microlens in each pixel requires additional processes. Moreover, curved CMOS image sensors (CISs) have been proposed by using tensile-stressed silicon wafers and flexible graphene [10,11]. The oblique incident light causes optical loss due to the field curvature of the module lens. Compared to the flat CIS, optical loss at the off-axis pixel was suppressed by overcoming curvature aberration. However, the process of curved wafers and graphene requires meticulous fabrications and is hard to be commercialized as of now. Furthermore, the inclined stacked layers were added on top of the microlenses [12]. The stacked layers had an anti-reflection layer, a high refractive index layer, and a low refractive index layer. Oblique incident light entering the microlens was controlled by using designed layers. However, shrinking uneven layers is a challenge for manufacturing technology.

Shifting optical stacks was suggested as one of the widely used methods to minimize the optical loss of off-axis pixels [13]. In the off-axis pixel without aligning the optical stacks, the microlens focuses on the periphery of the photodiode and adjacent pixels due to the obliquely incident light. Upon shifting the position of the microlens, the focus of the oblique incident light was able to be changed. Moreover, obliquely incident light causes unwanted absorption in the color filter. A shifted color filter was able to resolve the unwanted absorption at the adjacent CF. Therefore, the shifted optical stacks guide the oblique incident light into the photodiode. To enhance the performance of off-axis pixels, the aligned optical stacks must be optimized according to CRA. Moreover, the shifted distance of the optical stacks increases as the CRA increases. Typically, the shift distance of the optical stacks is aligned by repeating the steps to move optical stacks to find the satisfactory position that has the best sensitivity and the least crosstalk [14,15]. In the case of fabricating CIS, designed pixels are simulated to confirm optical performance. However, the method that repeats to optimize aligning the optical stacks must often be devoted to finding the exact position in the simulation. Performance verification time is significantly related to the rapid product launch and production cost. Therefore, a design rule aligning the optical stacks is needed for rapid research and product launch.

In order to save the developing time, the method to calculate the amount of optical stack shift was suggested [16]. The calculation includes the length between the microlens, CF, spacer metal layer, and so on as the parameters without a refractive index. However, the refractive index is required as one of the important factors to consider in the optical method. Thus, the optical stacks can be inaccurately aligned. In different methods, the calculations with the refractive index were proposed in order to align the only microlenses [17]. Therefore, occurred absorption in the CF is not considered and causes light loss. As another method, the optical stack is shifted and aligned integrally to find the position by using a calculation with the optical stacks’ average refractive index [18]. However, precise optical alignment is difficult because the average refractive index is used. Moreover, the calculation is adaptable for a frontside illumination (FSI) structure to have the interconnection metal layer on top of the silicon. For the high optical performance, most CISs use a backside illumination (BSI) structure that has the interconnection metal layer under silicon for high sensitivity [19]. In the case of the latest pixels, the deep trench isolation (DTI) and metal grid between each CF and PL have been widely used for eliminating optical crosstalk [20,21,22]. Moreover, the quad CF array, 2 × 2 microlens (2 × 2 ML), and in-pixel DTI have emerged as elements of advanced pixels [23,24,25]. Therefore, methods for aligning the optical stacks need to be defined according to the pixel structures’ development.

In this paper, a numerical method was proposed for aligning the optical stacks of pixels with the 2 × 2 ML, quad CF array, and in-pixel DTI. The suggested method can be applied to all pixels in the image sensor, regardless of CRA. In order to accurately align the optical stacks, the microlens and CF were separately shifted. Moreover, the optical stacks were aligned in order to enter the center of the in-pixel DTI to evenly distribute the oblique incident light. In the process of CIS production and simulation, the repeated process of shifting the microlens and CF as being time-consuming was able to be eliminated by using the suggested method. In Section 2, the suggested method is shown to find the position of the aligned optical stacks. Section 3 includes the simulation results and a discussion of the aligned optical stacks that depend on the changed pixel pitch and CRA. Section 4 presents the conclusions with the suggested method’s applicability.

## 2. Concept of the Numerical Method

**Figure 1:**
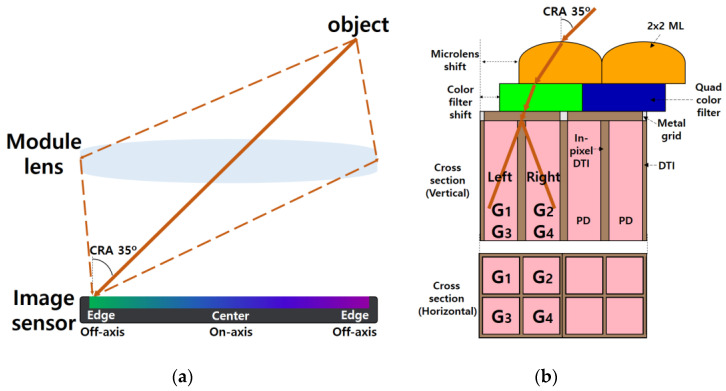
Schematic diagram of (**a**) the module lens and the image sensor, and (**b**) the cross sections of off-axis pixel at CRA 35°.

The incident light is focused on the image sensor through the module lens of a camera. Figure 1a shows a schematic diagram of the incident light coming from the module lens to the pixel at the edge of the image sensor. In this paper, we consider the CRA of the edge pixel as 35°. To minimize incident light loss, optical stacks must be aligned relative to the incident light at 35°. Figure 1b shows the cross sections of the off-axis pixel at CRA 35° with the aligned optical stacks. The pixels were composed of quad CF and 2 × 2 ML that share four pixels with the in-pixel DTI. The aligned optical stacks guide incident light to photodiodes (PDs) absorbing incident light. G1, G2, G3, and G4 indicate four photodiodes under the green color filter. Bayer CF has twice as many green pixels as red and blue pixels. Therefore, the optical stacks are usually aligned based on the green pixels.

In the case of the optical stacks, the significant factors are the microlens, CF, and PL. Among them, the focal point’s position and the transmitted light’s wavelength both change according to the movement of the microlens and CF. Imprecise optical alignment causes image quality to deteriorate. Therefore, the microlens and CF need to be individually and precisely moved. However, accurate alignment requires a great deal of effort and time.

In this paper, we propose a numerical method of the optical stack in the off-axis pixel of the image sensor. The shift amount, D, of the optical stack is determined by using Equation (1).

(1) $$D=\sqrt{h^{2}\left(\frac{1}{1-{\left(\frac{\sin\theta \times n_{1}}{n_{2}}\right)}^{2}}-1\right)}$$

The optical stack height, the refractive index of the optical stack, and the CRA are noted with *h*, $n_{2}$, and *θ*, respectively. The $n_{1}$ represents the refractive index of the material placed on top of the microlenses. A structure having additional stacked materials on top of microlens has been proposed. However, in the general case, it is filled with air with a refractive index of 1. Therefore, $n_{1}$ is used as a refractive index of 1 in this paper. The proposed method is based on Snell’s law, which is a fundamental principle of optics. Thus, the required elements are the refraction angle of the incident light and the refractive index of the material. Moreover, the height of each optical stack is required in order to extract the shift distance. High optical stacks cause a long optical path of oblique incident light to the photodiode and an increased shift distance.

**Figure 2:**
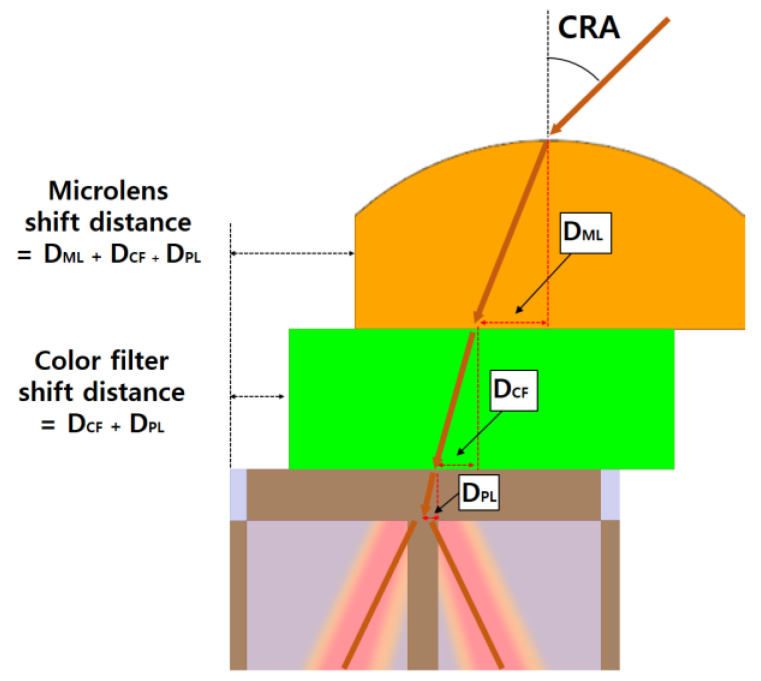
Extracted shift distance of microlens (ML) and CF from Equation (1) of the proposed method, which applies to the microlens, CF, and PL, respectively.

Figure 2 shows the pixels in which the optical stacks are aligned with the proposed numerical method. Oblique incident light passes through the microlens, CF, and PL to reach the in-pixel DTI. The in-pixel DTI was used to evenly distribute the light for off-axis pixels having quad CF array and 2 × 2 ML. Therefore, the aligned optical stacks must guide the oblique incident light to the in-pixel DTI in order to be distributed to the adjacent photodiode. The $D_{ML}$, $D_{CF}$, and $D_{PL}$ represent the microlens, CF, and PL calculation results from Equation (1), respectively. In the optical stacks, the positions of microlens and CF directly affect the incident light. To consider the transition between microlens and CF, the microlens and CF are individually shifted. The shift distance of the microlens is based on the path of the incident light passing through the microlens, CF, and PL. Therefore, the shift distance of the microlens is determined by the sum of $D_{ML}$, $D_{CF}$, and $D_{PL}$. Furthermore, the shift distance of CF is obtained as the sum of $D_{CF}$ and the $D_{PL}$. As the numerical method has CRA as a parameter, this method can be used for pixels at all positions of the image sensor. Moreover, the shift distance can be extracted, regardless of microlens and CF shapes, by the suggested method due to the parameter of the refractive index and height of the material. Therefore, the proposed method is expected to be effective in optical stack alignment.

## 3. Simulation Results and a Discussion

The effectiveness of the suggested numerical method was evaluated by using an optical simulator [26]. The finite-difference time-domain (FDTD) method is widely used to analyze optoelectronic devices. For the pixels used in the simulation, a quad CF array, 2 × 2 ML, and PL were used as the optical stacks. The heights of CF and PL are 0.6 and 0.2 μm, respectively. As pixel pitch, the simulated pixels have 1.0, 0.9, 0.8, 0.7, 0.6, and 0.5 μm. Moreover, the pixels have DTI, in-pixel DTI, and the tungsten grid. To confirm the effectiveness of the proposed numerical method, simulations were performed by changing the pixel pitch, CRA, microlens height, and radius of curvature (ROC). In this paper, the shift distance of CF is determined by applying Equation (1), which has parameters of refractive index, height, and CRA, to the CF and PL. As a result, the shift distance of the CF is 0.28 μm on the condition of CRA 35°.

**Figure 3:**
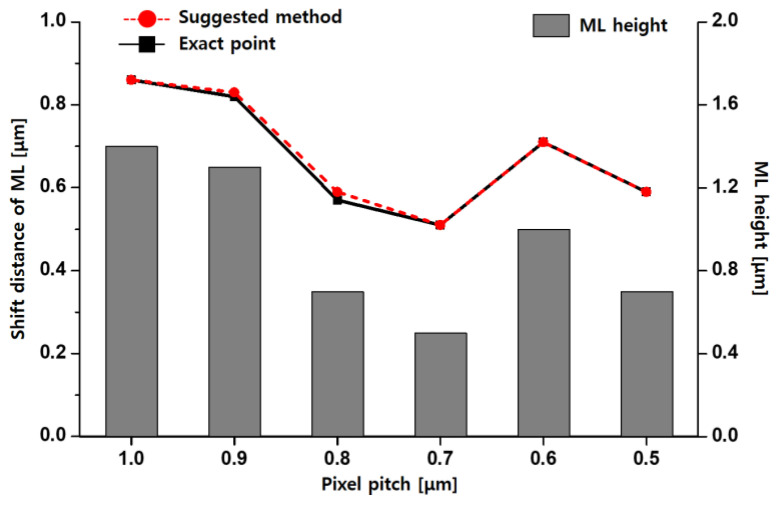
Comparison of simulation results and calculation results with the changed shift distance of microlens (ML) according to microlens height at CRA 35°.

First of all, Figure 3 shows the shift distance of the optical stack according to pixel pitch at CRA 35°. We compared the calculated results with the simulated results and the microlens heights. The exact point in the graph represents the simulated shift distance of the microlens when the adjacent pixels have uniform sensitivity. The suggested method represents the calculation results of the shift distance. The bar in the graph represents the microlens heights. The investigated pixel pitches were from 0.5 to 1.0 μm, and the microlens height and ROC were optimized for each pixel size on the condition of CRA 0°. The aligned optical stacks from the calculation using the proposed method were almost similar to the aligned pixel structure in the simulation. The relative error rate was up to 3.5% at the 0.8 μm pixel. In the suggested method, the calculation is based on Snell’s law. On the other hand, the FDTD method to solve Maxwell’s equation is applied in the optical simulation. Moreover, the simulation results are able to be slightly affected depending on the settings such as the size of the mesh, boundary condition, and so on. Therefore, the 0.9 and 0.8 μm pixels have some relative errors. However, the differences in the shift distance were negligible. From the simulation and calculation results, the microlens having a high height required more shifts for optical alignment. Therefore, the shift distance of the microlens was confirmed to be influenced by the height of the microlens.

**Figure 4:**
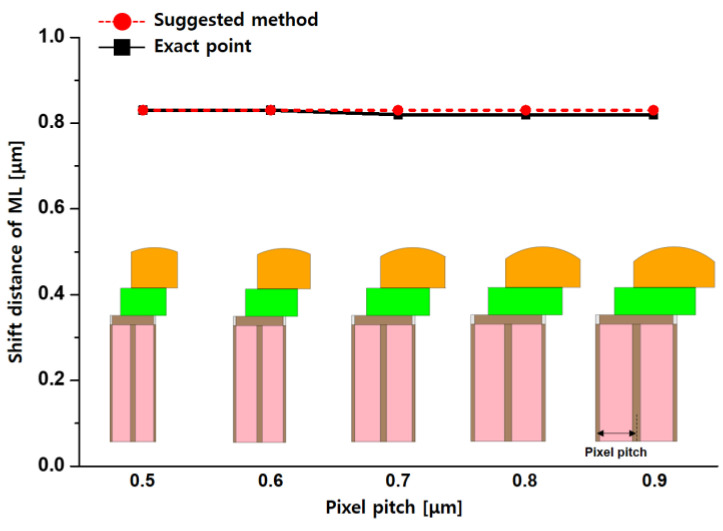
Comparison of simulation and calculation results with the varied pixel pitch from 0.9 μm to 0.5 μm on the same conditions of microlens (ML) height and ROC at CRA 35°.

Figure 4 shows comparison results with different pixel pitches at CRA 35°. All pixels have the same microlens height and ROC of 1.3 and 1.6 μm, respectively. According to the proposed calculation method, all pixels have the same shift distance as the optical stack in Figure 4 due to having the same parameters of refractive index, height, and CRA. Additionally, the shift distance of the exact point from the simulation was almost similar. The maximum relative error rates were confirmed as 1.21% at 0.7, 0.6, and 0.5 μm. The difference in the shift distance between the exact point and the suggested method was negligible: 0.01 μm. As the calculation and simulation results, the aligning optical stacks are independent of pixel pitch. Therefore, the pixel pitch is unnecessary for the calculation in the suggested method.

**Figure 5:**
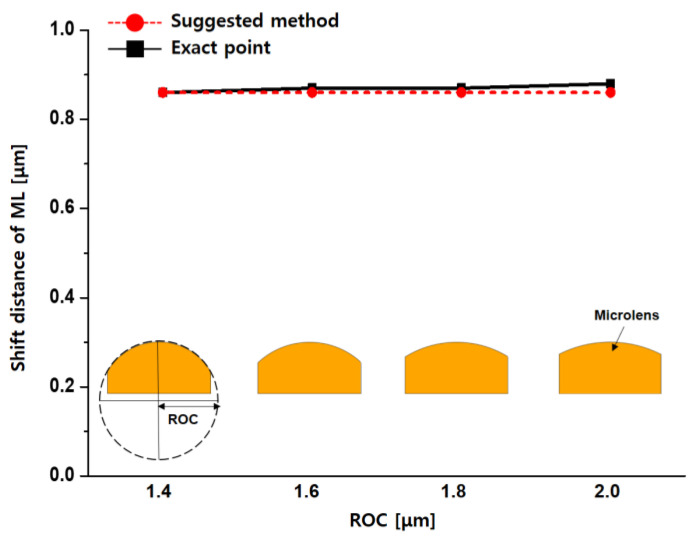
Comparison of simulation and calculation results with the changed microlens (ML) ROC from 1.4 to 2.0 μm on the same condition of pixel pitch and microlens height at CRA 35°.

The ROC of a microlens directly affects its focal point and focal length. Optimized microlens height and ROC are essential to improving pixel performance. On the other hand, the microlens ROC is excluded from Equation (1) in this paper. Therefore, the relationship between the ROC and the optical stack’s alignment is required in order to confirm the proposed method’s effectiveness. Figure 5 shows simulated pixel structures by changing only the ROC of the microlens. The simulated pixels have the same pixel pitch and microlens height as 1.0 and 1.4 µm, respectively. Compared to the exact point from the simulation, the maximum shift distance difference was 0.02 μm at the pixel having 2.0 μm of microlens ROC and the maximum relative error rate was insignificantly shown as 2.22%.

**Figure 6:**
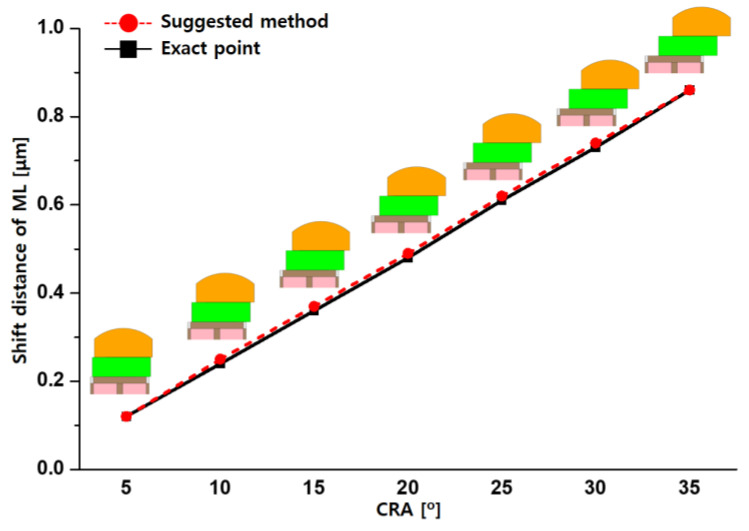
Comparison of simulation and calculation results with the changed CRA from 5° to 35° on the same condition of pixel pitch, microlens (ML) height, and ROC at CRA 35°.

The optical stacks are aligned by obliquely incident light, and the shift distance must be determined by the incident angle. Due to the changed CRA, according to the pixel position in the image sensor, the shift distance of the optical stacks is also changed. Therefore, CRA is one of the most important factors in the proposed method. Figure 6 shows the distance that the optical stack moved according to the CRA. In this simulation, pixel pitch, ROC, and microlens height were 1.0, 1.4, and 1.4 µm, respectively. Simulations were performed at 5° intervals for the CRA. For accurate simulation, the shift distance of not only the microlens, but also CF, was optimized based on the suggested method at all CRA. In the simulation result, the optimized shift distance of the microlens increases proportionally as the CRA increases. Between the exact point from the simulation and the suggested method by using Equation (1), the maximum relative error rate was shown as 4.17% at CRA 10°. However, the maximum shift distance difference was insignificant as it was only 0.01 μm. Therefore, the proposed method was confirmed as being suitable to apply all pixels in the image sensor, regardless of position.

**Figure 7:**
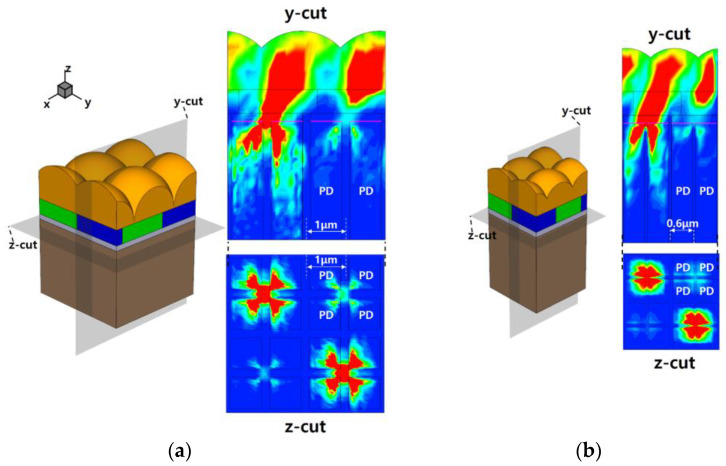
The simulated 3D structure pixel and beam profile of 2 × 2 ML pixel having pixel pitch of (**a**) 1.0 and (**b**) 0.6 μm at CRA 35°.

Simulation results were supplemented and visualized by beam profiles. Figure 7 shows the simulated 3D structure and beam profile of a 1.0 and 0.6 μm size pixel that was optically aligned by the proposed method at CRA 35°. The simulated pixels have optimized microlenses, as in Figure 2. Figure 7a shows the simulated 1.0 μm pixel structure with quad CF and 2 × 2 ML having a height of 1.4 μm and a ROC of 1.4 μm. The optical stacks, such as the microlens and CF, were aligned by using Equation (1). As a simulation result with calculated shift distance, the beam profile indicates even distributed oblique incident light to four pixels under the quad CF. In the simulation, the optical stacks were aligned by using the suggested method in order to guide incident light to adjacent pixels under the shared CF from in-pixel DTI. Therefore, the effectiveness of the suggested method could be confirmed in the optical path of the beam profile. Figure 7b shows the 2 × 2 ML pixel having a pixel pitch of 0.6 μm with a quad CF array. The microlens is optimized by the height of 1.0 μm and ROC of 0.9 μm. As a result, the beam profile shows the incident light separated into four pixels under the shared CF, as shown in Figure 7a. Therefore, the optical stack was almost precisely aligned from the calculation with the suggested method, showing its efficacy.

## 4. Conclusions

A numerical method was proposed for aligning the optical stacks at the image sensor’s off-axis pixel. The shift distance of the optical stacks can be extracted by using a suggested method with the parameters of optical stack height, refractive index, and CRA. Therefore, the shift distances for mediums are able to be found individually by using a numerical method. In this paper, the microlens and CF which are the most significant factors of optical stacks were individually shifted for optical alignment. An optical simulator was used to confirm the suggested method’s effectiveness. As an advanced pixel structure, the simulations were investigated on the condition of BSI sub-micron pixels having quad CF array, 2 × 2 ML, and in-pixel DTI. The simulated pixels have 1.0, 0.9, 0.8, 0.7, 0.6, and 0.5 μm as sub-micron. According to the proposed method, the microlens and CF were shifted individually and compared to the exact point that indicates the optimized alignment of the optical stack. Moreover, the correlation with the proposed structure was confirmed by changing the pixel pitch, microlens height, microlens ROC, and CRA. As a result of the simulation, the maximum difference in shift distance between the suggested method and the exact point was insignificant at 0.02 μm, and the relative error rate was 3.5% in the pixel with the optimized microlens shape for high sensitivity. The parameters of the proposed method were significantly affected by the optical stack height and CRA. On the other hand, pixel pitch and microlens ROC were almost unrelated to the optical stack’s shift distance. The optical stacks aligned by calculating using the proposed method were matched or almost similar to the optimized point in the simulation. Therefore, we demonstrated that a numerical method having parameters such as a refractive index, the height of the optical stack, and CRA is suitable for aligning the optical stacks in the off-axis pixel. In the case of misaligned optical stacks due to some relative error rate of the suggested method, optimized optical stack alignment can be extracted as additional simulations without much time due to the proposed method serving as an initiating point for aligned optical stacks.

In the image sensor’s off-axis pixel, the oblique incident light from the module lens causes unwanted optical loss. Therefore, a misaligned optical stack degrades image quality. For fabricating CIS, the simulation was used to confirm the optical performance of designed pixels. However, a lot of time has been devoted to finding different optimized alignments depending on the off-axis pixel position in the image sensor. Performance verification time is one of the significant factors in production cost and rapid product launch. As a solution, the proposed numerical method is expected to contribute to reducing verification time and cost in the image sensor market. The proposed numerical method was satisfactorily applied in the simulation. As future work, we consider that it is necessary to investigate the correlation between a proposed numerical method and produced image sensor to confirm the effectiveness of the suggested method. Although the meticulous fabrication of aligned optical stacks is required, the numerical method provides the benefits of reducing device development time and cost through the demonstration of effectiveness.

## Data Availability

Not applicable.
